# PPAR-α Agonist Fenofibrate Prevented Diabetic Nephropathy by Inhibiting M1 Macrophages via Improving Endothelial Cell Function in db/db Mice

**DOI:** 10.3389/fmed.2021.652558

**Published:** 2021-06-29

**Authors:** Xiaomeng Feng, Xia Gao, Shuo Wang, Mengxiu Huang, Zhencheng Sun, Hengbei Dong, Haitian Yu, Guang Wang

**Affiliations:** ^1^Department of Endocrinology, Beijing Chao-Yang Hospital, Capital Medical University, Beijing, China; ^2^Department of Infectious Diseases, Beijing Traditional Chinese Medical Hospital, Capital Medical University, Beijing, China; ^3^Department of Hepatobiliary, Beijing Chao-Yang Hospital, Capital Medical University, Beijing, China; ^4^Department of Osteology, Beijing Chao-Yang Hospital, Capital Medical University, Beijing, China; ^5^Department of Reproductive Medicine, Beijing Obstetrics and Gynecology Hospital, Capital Medical University, Beijing, China; ^6^Education Division, Beijing Chao-Yang Hospital, Capital Medical University, Beijing, China

**Keywords:** PPAR-α agonists, diabetic nephropathy, macrophages, endothelial function, HIF-1α, Notch1

## Abstract

**Background:** Diabetic nephropathy (DN) is one of the major diabetic microvascular complications, and macrophage polarization plays a key role in the development of DN. Endothelial cells regulate macrophage polarization. Peroxisome proliferator-activated receptor (PPAR)-α agonists were demonstrated to prevent DN and improve endothelial function. In this study, we aimed to investigate whether PPAR-α agonists prevented DN through regulating macrophage phenotype via improving endothelial cell function.

**Methods:** Eight-week-old male C57BLKS/J db/m and db/db mice were given fenofibrate or 1% sodium carboxyl methylcellulose by gavage for 12 weeks.

**Results:** Db/db mice presented higher urinary albumin-to-creatinine ratio (UACR) than db/m mice, and fenofibrate decreased UACR in db/db mice. Fibrosis and collagen I were elevated in db/db mouse kidneys compared with db/m mouse kidneys; however, they were decreased after fenofibrate treatment in db/db mouse kidneys. Apoptosis and cleaved caspase-3 were enhanced in db/db mouse kidneys compared to db/m mouse kidneys, while fenofibrate decreased them in db/db mouse kidneys. Db/db mice had a suppression of p-endothelial nitric oxide synthase (eNOS)/t-eNOS and nitric oxide (NO), and an increase of angiopoietin-2 and reactive oxygen species (ROS) in kidneys compared with db/m mice, and fenofibrate increased p-eNOS/t-eNOS and NO, and decreased angiopoietin-2 and ROS in db/db mouse kidneys. Hypoxia-inducible factor (HIF)-1α and Notch1 were promoted in db/db mouse kidneys compared with db/m mouse kidneys, and were reduced after fenofibrate treatment in db/db mouse kidneys. Furthermore, the immunofluorescence staining indicated that M1 macrophage recruitment was enhanced in db/db mouse kidneys compared to db/m mouse kidneys, and this was accompanied by a significant increase of tumor necrosis factor (TNF)-α and interleukin (IL)-1β in kidneys and in serum of db/db mice compared with db/m mice. However, fenofibrate inhibited the renal M1 macrophage recruitment and cytokines associated with M1 macrophages in db/db mice.

**Conclusions:** Our study indicated that M1 macrophage recruitment due to the upregulated HIF-1α/Notch1 pathway induced by endothelial cell dysfunction involved in type 2 diabetic mouse renal injury, and PPAR-α agonist fenofibrate prevented DN by reducing M1 macrophage recruitment via inhibiting HIF-1α/Notch1 pathway regulated by endothelial cell function in type 2 diabetic mouse kidneys.

## Introduction

Diabetic nephropathy (DN), the main cause of end-stage renal disease, is one of the major microvascular complications of diabetes. The primary initiating mechanism in DN is hyperglycemia-induced vascular dysfunction (1). Nitric oxide (NO) is a major regulator of vascular tone. Diminution of NO has been considered as a major mechanism underlying development of diabetic complications involving the vasculature, especially DN (2). There are three distinct genes that encode three nitric oxide synthase (NOS) isoforms: neuronal nitric oxide synthase (nNOS), inducible nitric oxide synthase (iNOS), and endothelial nitric oxide synthase (eNOS). Both nNOS and iNOS are weakly expressed in the kidney. Most eNOS is strongly expressed in renal endothelial cells, although tubular expression of eNOS also occurs (3). Thus, NO in kidneys is mainly generated by eNOS in endothelial cells. Recent studies have found that eNOS and NO were decreased, whereas iNOS and nNOS were increased in diabetic rats (4, 5). ENOS dysfunction in endothelial cells has been demonstrated to have a key role in the development of DN. Both type 1 and type 2 diabetic mice with eNOS deficiency are more susceptible to renal injury compared to wild type diabetic mice (6–8).

Renal vasoconstriction, induced by the deficiency of NO, likely contributes to renal injury due to renal tissue hypoxia, which leads to the increased expression of hypoxia-inducible factor (HIF)-1α. Renal vasodilatation function was improved through upregulating the activity of eNOS and consequently downregulating the expression of HIF-1α in septic shock rats, which could be antagonized by eNOS inhibitor (9), supporting that the activation of eNOS could regulate the level of HIF-1α. Our previous study has found that increased angiopoietin-2 (Ang-2), an indicator of endothelial dysfunction, was related to elevated HIF-α in mouse kidneys (10). Recent studies have also proven that the inhibition of HIF-1 protected against DN (11). Our previous study showed that endothelial-specific prolyl hydroxylase domain protein-2 (PHD2) knockout (PHD2ECKO) mice, with the upregulated expression of HIF-α in endothelial cells due to the deficient degradation of HIF, presented significant renal fibrosis through activating Notch (10).

Notch is a key regulator of cellular development, differentiation, survival and function, which is usually achieved by interacting with other pathways, including HIF-1α signaling. HIF-1α, which is induced by hypoxia, contributes to Notch increasing (12), and inhibiting HIF-1α decreases Notch activity (13). Notch signaling promotes the development of DN including accelerating pathological changes in glomerulus, tubules, interstitium, and blood vessels (14). Moreover, Notch has been proven to regulated macrophage polarization which further induces fibrosis in DN (15).

Accumulating evidence suggests the critical role of macrophage polarization in the development of fibrosis, and the effect of M1 macrophage polarization on accelerating renal fibrosis in DN (1, 15–17). In the progression of DN, monocytes are rapidly recruited to sites of diabetic complications and differentiate into macrophages, which leads to diabetic nephropathy, fibrosis, and proteinuria. Macrophage polarization can be regulated at least partially by endothelial cells. Endothelial-specific Ang-2 overexpressed mice showed increased macrophage infiltration (18, 19). Endothelial cells decrease M1 marker expression (20). Furthermore, endothelial cell senescence was in connection with renal M1 macrophage accumulation (21). These researches have suggested that endothelial cell function might regulate M1 macrophage accumulation in kidneys. Recent researches have found that hypoxia regulated macrophage polarization, and there was a significant relationship between HIF-1α and M1 macrophage polarization (22, 23). The regulation effects of Notch on macrophage polarization and fibrosis have been paid generally attention in renal injury (1, 15–17). Notch can promote renal fibrosis through inducing M1 macrophage polarization. However, whether endothelial function regulates macrophage polarization via HIF-1α/Notch1 pathway has been uncertain.

Peroxisome proliferator-activated receptor (PPAR)-α is a member of the nuclear hormone receptor superfamily of ligand-activated transcription factors and plays an important role in lipid metabolism (24). The consistency of clinical data from PPAR-α agonists studies have demonstrated consistent benefit with fenofibrate on preventing the progression of diabetic microvascular diseases, independent of lipid levels (25). The FIELD study showed significant beneficial effects on diabetic complications in micro-vascular (i.e., nephropathy, retinopathy, and non-traumatic amputations) (26). The DAIS study indicated that fenofibrate prevented the progression to microalbuminuria on a long-term basis in diabetic patients (27). Furthermore, our previous research also found that fenofibrate reduced microalbuminuria in patients with type 2 diabetes (28). In addition, PPAR-α agonists have been demonstrated to prevent DN and reduce proteinuria in both type 1 and type 2 diabetic animals (29–32). Nevertheless, the mechanism of how PPAR-α agonist fenofibrate prevents DN has not been fully explored.

In our previous studies, high glucose induced endothelial dysfunction as indicated by an increased reactive oxygen species (ROS) generation and a decreased NO production in human umbilical vein endothelial cells (HUVECs) (33). However, fenofibrate recoupled eNOS and increased the secretion of NO in HUVECs (34). In addition, fenofibrate significantly improved coronary flow velocity reserve (CFVR) and arterial stiffness in patients with hypertriglyceridemia (35). Our previous results suggested that PPAR-α agonist fenofibrate could adjust endothelial function and vascular tone. In addition, studies *in vivo* and *vitro* have found that PPAR-α agonists had therapeutic effects on ischemic retina diseases, especially on diabetic retinopathy, through the downregulation of HIF-1α in endothelial cells (36, 37). PPAR-α agonists diminished hypoxia-induced HIF-1α expression and activity in cancer cells (38). Moreover, upregulating PPAR-α could suppress Notch-1 signaling (39).

PPAR-α agonists prevented renal fibrosis in DN (40). However, the effects of PPAR-α agonists on macrophage phenotype have been still unclear. Furthermore, whether PPAR-α agonists prevent DN through regulating macrophage phenotype through HIF-1α/Notch1 pathway adjusted by endothelial cell function has not been studied yet. In this study, we aimed to investigate the mechanism of preventing DN by PPAR-α agonist fenofibrate.

## Materials and Methods

The animal experiments were approved by the Animal Ethics Committee of Beijing Chao-Yang Hospital, Capital Medical University and were performed in accordance with animal care guidelines of Beijing Chao-Yang Hospital, Capital Medical University.

### Experimental Animal Model and Treatment

Seven-week-old male C57BLKS/J db/m and db/db mice (t002407) were obtained from Nanjing Biomedical Research Institute of Nanjing University, Nanjing, China. They were divided into four groups, db/m group, db/m+F group, db/db group, and db/db+F group.

Db/m+F and db/db+F groups (*n* = 6 for each group) were given 100 mg/kg of fenofibrate (0.1%, w/w, Sigma, St Louis, MO, USA) dissolved in 1% sodium carboxyl methylcellulose (Na-CMC) by gavage once per day for 12 weeks starting at 8 weeks of age. Db/m and db/db groups (*n* = 6 for each group) were treated with 1% Na-CMC alone by gavage once per day for 12 weeks starting at 8 weeks of age (29).

All mice were housed in clear cages (*n* = 3/cage) and maintained on a 12-h light/dark cycle (lights on 08:00–20:00 h) at 22 ± 1°C with water and food available *ad libitum*. After 12-week administration, all mice were placed in metabolic cages separately. At week 20, all animals were anesthetized by intraperitoneal injection of a mixture of Rompun 10 mg/kg (Bayer Korea, Ansan, Gyeonggi-Do, Korea) and Zoletil 30 mg/kg (Virbac, Carros, France). Blood was obtained from the left ventricle and was stored at −80°C for subsequent analyses. The mouse kidneys were removed.

### Measurements of Blood and Urinary Parameters

Blood was collected following an overnight fast for 12 h. A 24-h urine collection was obtained using metabolic cages. Blood glucose (GLU) level was detected by HemoCue B-Glucose kit (HemoCue AB, Angelholm, Sweden). Insulin (INS) level was detected by radioimmunoassay kit (Linco Reasearch, St Charles, MO, USA). Triglycerides (TG) and total cholesterol (TC) concentrations were measured by an auto-analyzer (Wako, Osaka, Japan). Blood urea nitrogen (BUN) was measured by iStat-Kit (HESKA, Fort Collins, MO, USA). Serum and urine creatinine concentrations were detected by HPLC (Beckman Instruments, Fullerton, CA, USA). Urinary albumin concentration was detected using an immunoassay (Bayer, Elkhart, IN, USA). Urinary albumin-to-creatinine ratio (UACR) was calculated as urine albumin/urine creatinine (μg/mg).

### Light Microscopic Study

The renal tissues were fixed in neutral-buffered 10% formalin solution (SF93-20; Fisher Scientific, Pittsburgh, PA, USA). The histology was measured by Hematoxylin & Eosin (H&E) staining (ab245880, Abcam, Cambridge, MA, USA) and Periodic Acid Schiff (PAS) staining (ab150680, Abcam, Cambridge, MA, USA). The fibrosis score was based on the ratio of fibrotic area to total area determined by Sirius red staining (ab150686, Abcam, Cambridge, MA, USA) and Masson's trichrome staining (ab150686, Abcam, Cambridge, MA, USA). Renal apoptosis was detected by TUNEL (MK1018, Boster, Wuhan, China). The renal samples were also embedded in frozen optimal cutting temperature compound (4585; Fisher Health Care, Houston, TX, USA). Frozen sections were prepared (8 μm in thickness). Reactive oxygen species (ROS) in frozen sections was measured by dihydroethidium (DHE) staining. Immunostaining of F4/80 (1:100; Abcam, Cambridge, MA, USA), CD86 (1:100; R&D systems, MN, USA), and CD32/16 (1:100; R&D systems, MN, USA) antibodies were in fresh frozen sections. These immune-stained sections were incubated with donkey anti-rat IgG-FITC (Sigma-Aldrich, Shanghai, China) (1:500) or anti-goat IgG-Cy3 produced in rabbit (Sigma-Aldrich, Shanghai, China) (1:500). For the quantification of proportional areas of staining, 10 areas were used, which were randomly located in mouse kidneys. Image J software (NIH, Bethesda, MD, USA) was used for image-analysis.

### Western Blot Analyses

The renal cortex tissues were homogenized in lysis buffer. The homogenates were centrifuged at 16,000 × g at 4°C for 10 min. A bicinchoninic acid protein assay kit (Pierce Co, Rockford, IL, USA) was used for measuring the protein concentrations. Equal amounts (20 μg) of the protein were separated by 10% sodium dodecyl sulfate polyacrylamide gel electrophoresis and transferred to a polyvinylidene difluoride (PVDF) membrane. The membranes were blocked with 5% non-fat dry milk in Tris-buffered saline and were incubated by the following primary antibodies overnight: podocin (1:1,000; Sigma-Aldrich, Shanghai, China), collagen I (1:1,000; Abcam, Cambridge, MA, USA), cleaved caspase-3 (1:1,000; Abcam, Cambridge, MA, USA), phospho-endothelial nitric-oxide synthase (p-eNOS) (1:1,000; BD transduction, San Jose, CA, USA), total-endothelial nitric-oxide synthase (t-eNOS) (1:1,000; BD transduction, San Jose, CA, USA), Ang-2 (1:1,000; Santa Cruz, CA, USA), HIF-1α (1:1,000; Novus Bio, Littleton, CO, USA), Notch1 (1:1,000; Abcam, Cambridge, MA, USA), and β-actin (1:1,000; Cell Signaling, Danvers, MA, USA). After washing, the membranes were incubated for 2 h with an anti-rabbit or anti-mouse secondary antibody coupled to horseradish peroxidase (1:5,000; Santa Cruz, CA, USA). Luminol was used as substrate. Densitometric analyses were performed using image acquisition and analysis software (Bio-Rad).

### RNA Extraction and Quantitative Reverse Transcriptase PCR (RT-PCR)

The total RNA was extracted from mouse renal tissue using TRIzol (Invitrogen, Carlsbad, CA, USA). RT -PCR was executed with QuantiTect SYBR Green PCR Kit (Qiagen, Valencia, CA). Primer sequences of tumor necrosis factor (TNF)-α were 5′–CAG GAG GGA GAA CAG AAA CTC CA−3′ (sense) and 5′–CCT GGT TGG CTG CTT GCT T−3′ (antisense), primer sequences of IL-1β were 5′–GCA ACT GTT CCT GAA CTC AAC T−3′ (sense) and 5′–ATC TTT TGG GGT CCG TCA ACT−3′ (antisense), and primer sequences of β-actin were 5′-CAT CCG TAA AGA CCT CTA TGC CAA C−3′ (sense) and 5′- ATG GAG CCA CCG ATC CAC A−3′ (antisense).

### NO Levels

Renal NO production was measured using commercial kits (Sigma-Aldrich, Shanghai, China), performed in accordance with the manufacturer's protocol.

### Serum Inflammatory Cytokines

Serum inflammatory cytokines associated with M1 macrophages, TNF-α and interleukin (IL)-1β, were measured with ELISA (eBioscience, San Diego, CA, USA). All assays were performed according to the manufacturer's protocol.

### Statistical Analyses

All data were analyzed using Statistical Package for Social Sciences version 22.0 (SPSS, Inc., Chicago, IL, USA). Data were expressed as means ± S.E.M. Comparisons of the means of corresponding values in four groups were performed using one-way ANOVA. All tests were two-sided, and a *P* < 0.05 was used to indicate statistical significance for the results.

## Results

### Physical and Biochemical Characteristics

**Figure 1:**
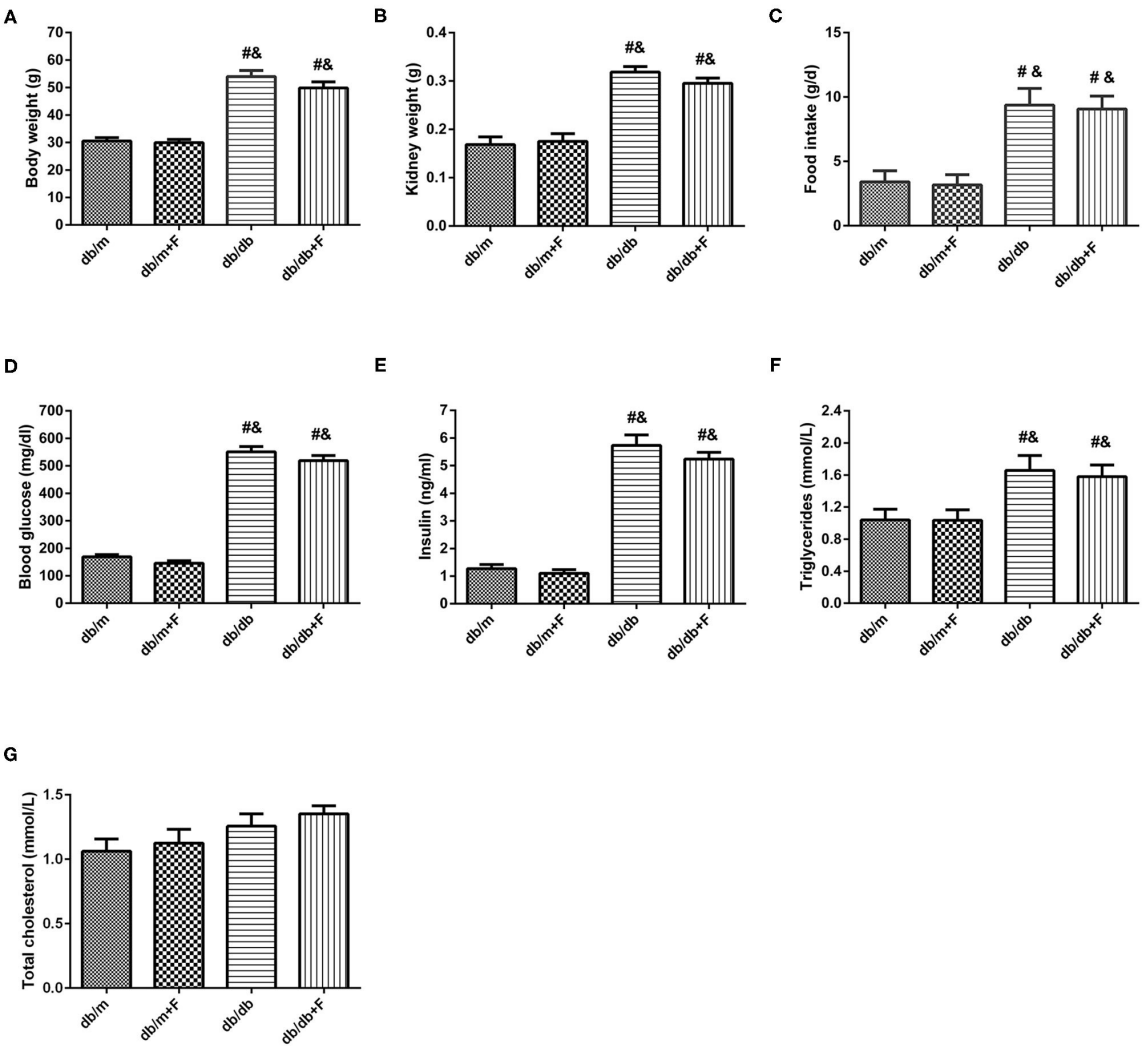
Physical and biochemical characteristics in db/m, db/m+F, db/db, and db/db+F groups. **(A)** Body weight. **(B)** Kidney weight. **(C)** Food intake. **(D)** Fasting blood glucose level. **(E)** Fasting Insulin level. **(F)** Fasting triglycerides. **(G)** Fasting total cholesterol. *n* = 6 mice/group. ^#^*P* < 0.05, vs. db/m group; ^&^*P* < 0.05 vs. db/m+F group. Db/m, db/m mice without fenofibrate treatment; db/m+F, db/m mice with fenofibrate treatment; db/db, db/db mice without fenofibrate treatment; db/db+F, db/db mice with fenofibrate treatment. Data are means ± S.E.M.

As presented in Figure 1, body weight (BW), kidney weight (KW), food intake, blood glucose (GLU), insulin (INS), and triglycerides (TG) were significantly higher for db/db and db/db+F groups than db/m and db/m+F groups. Db/m and db/m+F groups were similar in BW, KW, food intake, GLU, INS, and TG, and there was no difference in BW, KW, food intake, GLU, INS, and TG between db/db and db/db+F groups (Figures 1A–F). Moreover, the mice in four groups were similar in total cholesterol (TC) (Figure 1G).

### Renal Phenotype

**Figure 2:**
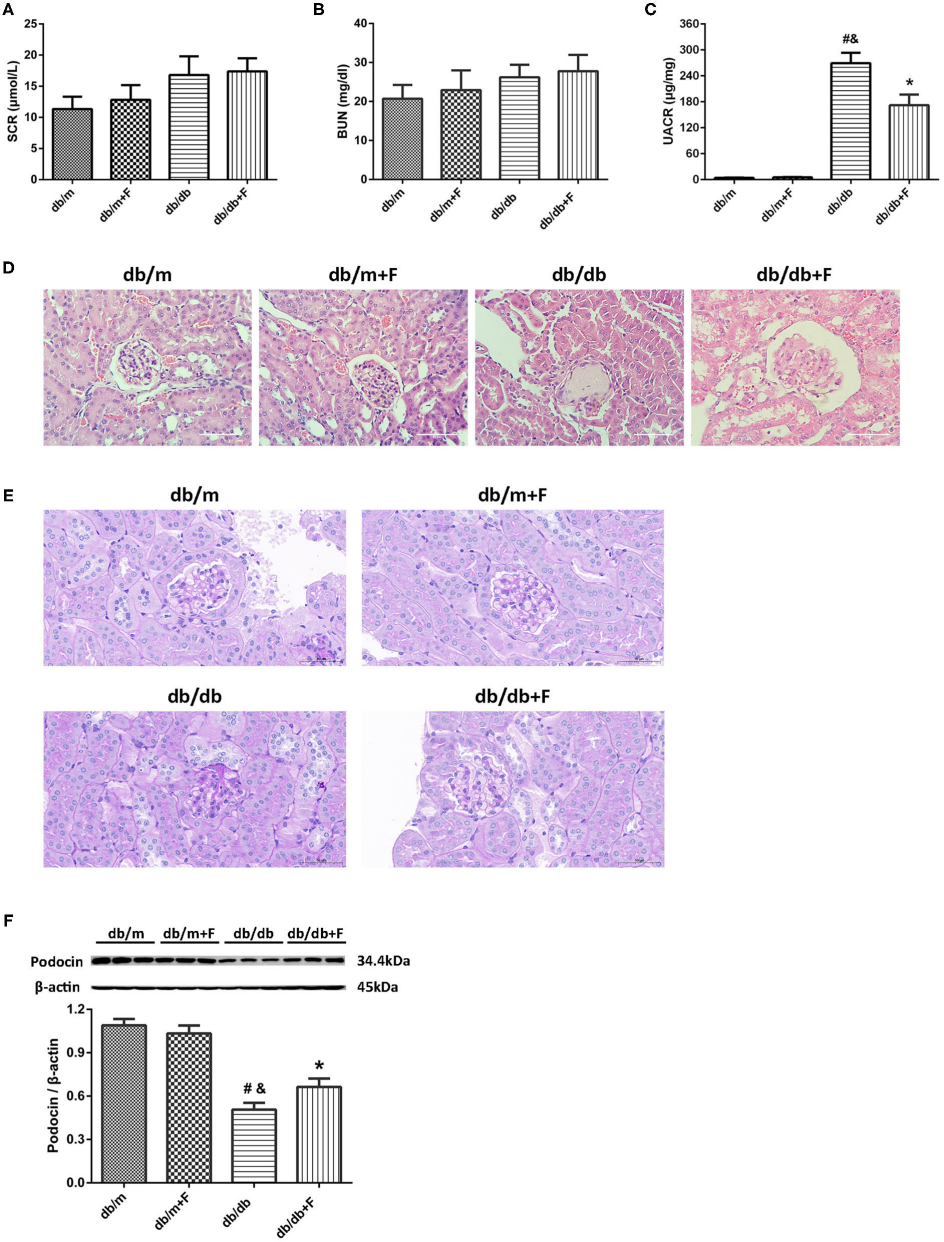
Renal phenotype in db/m, db/m+F, db/db, and db/db+F groups. **(A)** Serum creatinine (SCr). **(B)** Blood urea nitrogen (BUN). **(C)** Urinary albumin-to-creatinine ratio (UACR). **(D)** Representative photographs of mouse kidneys by H&E staining. **(E)** Representative photographs of mouse kidneys by PAS staining. **(F)** Representative photographs and quantification of podocin in mouse kidneys detected by western blot. *n* = 6 mice/group. ^#^*P* < 0.05 vs. db/m group; ^&^*P* < 0.05 vs. db/m+F group; *P < 0.05 vs. db/db group. Db/m, db/m mice without fenofibrate treatment; db/m+F, db/m mice with fenofibrate treatment; db/db, db/db mice without fenofibrate treatment; db/db+F, db/db mice with fenofibrate treatment. Data are means ± S.E.M.

As shown in Figure 2, no significant difference was observed in serum creatinine (SCr) and blood urea nitrogen (BUN) of all mice (Figures 2A,B). Db/db group presented higher UACR level than db/m and db/m+F groups. However, db/db+F group had the significantly decreased level of UACR compared with db/db group (Figure 2C). H&E staining and PAS staining indicated that mice in db/db group presented glomerular mesangial expansion and glomerulosclerosis in kidneys, but fenofibrate improved these changes in kidneys of db/db+F group (Figures 2D,E). In addition, western blot indicated that renal podocyte marker-podocin was decreased in db/db group compared with db/m and db/m+F groups, and was increased in db/db+F group compared with db/db group (Figure 2F). These findings indicated that PPAR-α agonist fenofibrate prevented DN in type 2 diabetic mice.

### Renal Fibrosis

**Figure 3:**
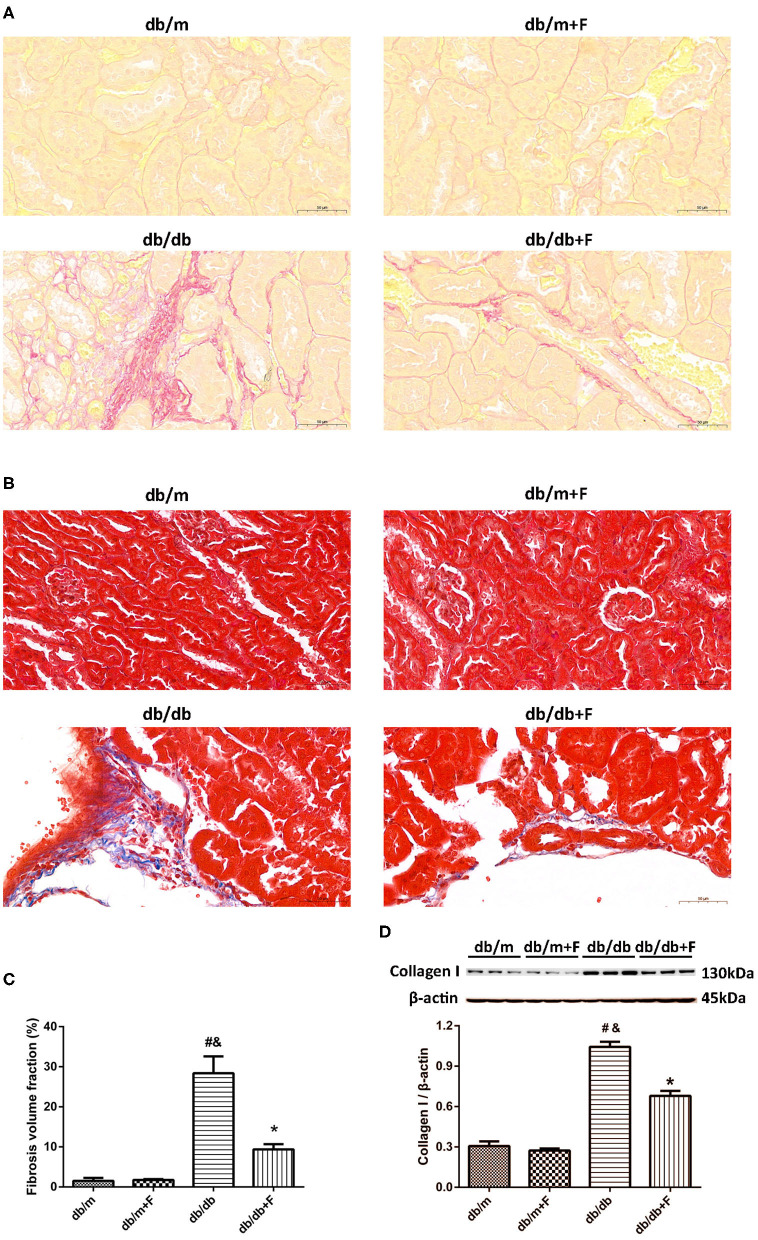
Renal fibrosis in db/m, db/m+F, db/db, and db/db+F groups. **(A–C)** Representative photographs and quantification of renal fibrosis measured by Sirius red staining **(A)** and Masson's staining **(B)**. **(D)** Representative photographs and quantification of collagen I in mouse kidneys detected by western blot. *n* = 6 mice/group. ^#^*P* < 0.05 vs. db/m group; ^&^*P* < 0.05 vs. db/m+F group; *P < 0.05 vs. db/db group. Db/m, db/m mice without fenofibrate treatment; db/m+F, db/m mice with fenofibrate treatment; db/db, db/db mice without fenofibrate treatment; db/db+F, db/db mice with fenofibrate treatment. Data are means ± S.E.M.

Next, we examined whether fenofibrate alleviated renal fibrosis in diabetic mice. Sirius red staining and Masson's staining exhibited that renal fibrosis was promoted in db/db group compared to db/m and db/m+F groups, but was alleviated in db/db+F group compared with db/db group (Figures 3A–C). Western blot further indicated that collagen I was enhanced in the kidneys of db/db group compared to db/m and db/m+F groups, but was inhibited in the kidneys of db/db+F group compared with db/db group (Figure 3D). These findings suggested that there was more significant renal fibrosis in type 2 diabetic mice than non-diabetic mice, and fenofibrate would prevent renal fibrosis in type 2 diabetic mice.

### Renal Apoptosis

**Figure 4:**
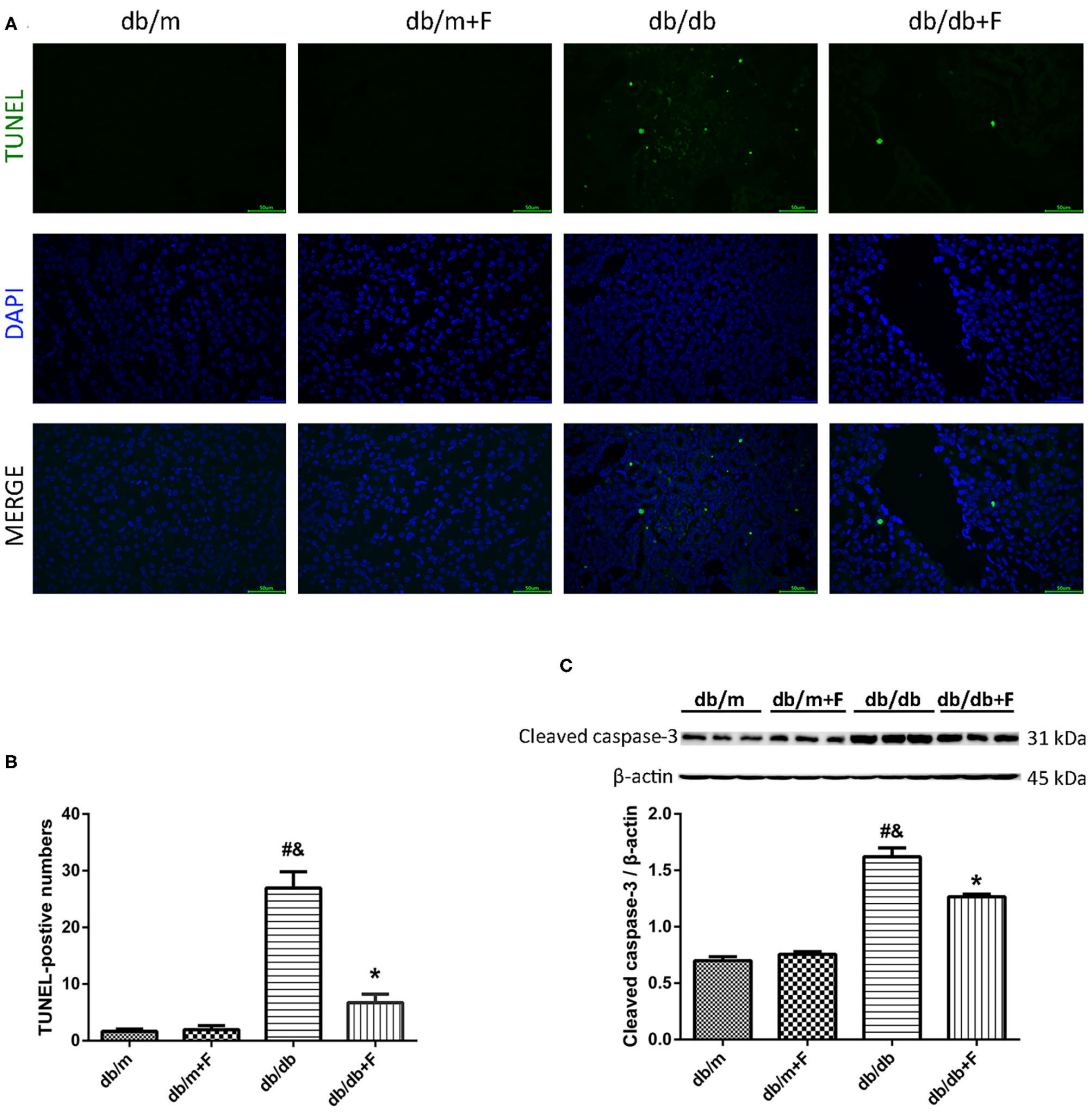
Renal apoptosis in db/m, db/m+F, db/db, and db/db+F groups. **(A,B)** Representative photographs and quantification of renal apoptosis measured by TUNEL assay. **(C)** Representative photographs and quantification of cleaved caspase-3 in mouse kidneys detected by western blot. *n* = 6 mice/group. ^#^*P* < 0.05 vs. db/m group; ^&^*P* < 0.05 vs. db/m+F group; *P < 0.05 vs. db/db group. Db/m, db/m mice without fenofibrate treatment; db/m+F, db/m mice with fenofibrate treatment; db/db, db/db mice without fenofibrate treatment; db/db+F, db/db mice with fenofibrate treatment. Data are means ± S.E.M.

Then, we detected mouse renal apoptosis. As shown in Figure 4, TUNEL assay showed that renal apoptosis was increased in db/db group compared to db/m and db/m+F groups, whereas renal apoptosis was decreased in db/db+F group compared with db/db group (Figures 4A,B). Moreover, western blot indicated that cleaved caspase-3 was promoted in kidneys of db/db group compared with db/m and db/m+F groups, and was inhibited in kidneys of db/db+F group compared with db/db group (Figure 4C). These findings indicated that type 2 diabetes enhanced apoptosis in mouse kidneys, but PPAR-α agonist fenofibrate treatment prevented apoptosis in mouse kidneys of type 2 diabetes.

### Endothelial Function in Mouse Kidneys

**Figure 5:**
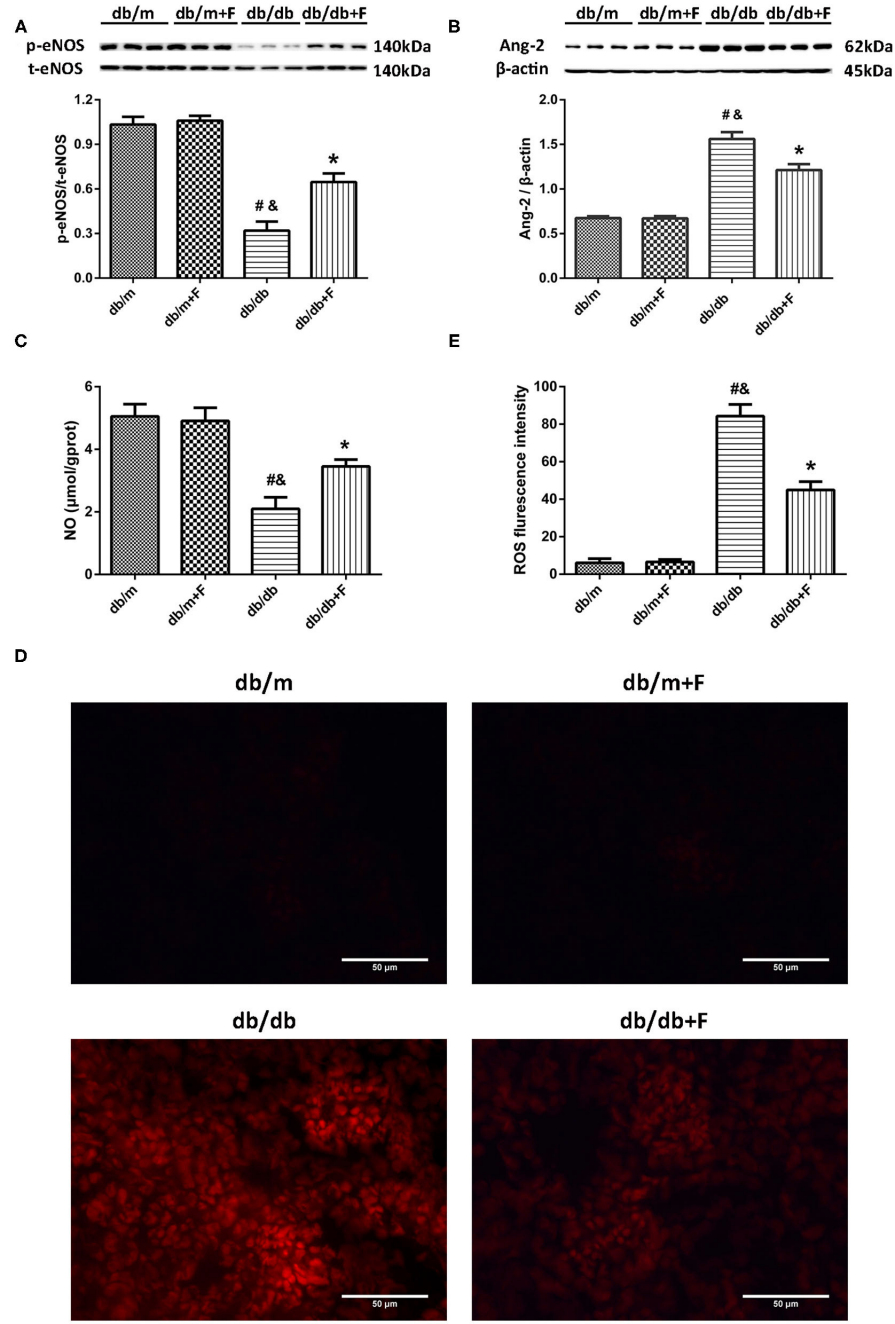
Endothelial function in mouse kidneys. **(A)** Representative photographs and quantification of phospho-endothelial nitric-oxide synthase (p-eNOS)/total-endothelial nitric-oxide synthase (t-eNOS) in mouse kidneys measured by western blot. **(B)** Representative photographs and quantification of angiopoietin-2 (Ang-2) in mouse kidneys measured by western blot. **(C)** Quantification of nitric oxide (NO) level in mouse kidneys measured by Griess. **(D,E)** Representative photographs and quantification of reactive oxygen species (ROS) formation in mouse kidneys by dihydroethidium staining. *n* = 6 mice/group. ^#^*P* < 0.05 vs. db/m group; ^&^*P* < 0.05 vs. db/m+F group; *P < 0.05 vs. db/db group. Db/m, db/m mice without fenofibrate treatment; db/m+F, db/m mice with fenofibrate treatment; db/db, db/db mice without fenofibrate treatment; db/db+F, db/db mice with fenofibrate treatment. Data are means ± S.E.M.

Accumulating evidence suggests an involvement of endothelial dysfunction in the diabetic renal injury (6–8), and our previous study demonstrated that fenofibrate improved endothelial function (34). In current study, western blot showed that a significant suppression of p-eNOS/t-eNOS and a significant increase of Ang-2 in kidneys of db/db group compared to db/m and db/m+F groups, but fenofibrate treatment improved p-eNOS/t-eNOS and Ang-2 in kidneys of db/db+F group (Figures 5A,B). This was accompanied by a significant reduction of NO in renal tissues of db/db group compared to db/m and db/m+F groups, which was also improved by fenofibrate treatment in renal tissues of db/db+F group (Figure 5C). In addition, DHE staining showed that ROS formation was increased in renal tissues of db/db group compared to db/m and db/m+F groups, while ROS formation was decreased in db/db+F group compared to db/db group (Figures 5D,E). These results indicated that there was more significant endothelial dysfunction in type 2 diabetic mouse kidneys compared to non-diabetic mouse kidneys, and fenofibrate improved endothelial function in type 2 diabetic mouse kidneys.

### HIF-1α and Notch1 in Mouse Kidneys

**Figure 6:**
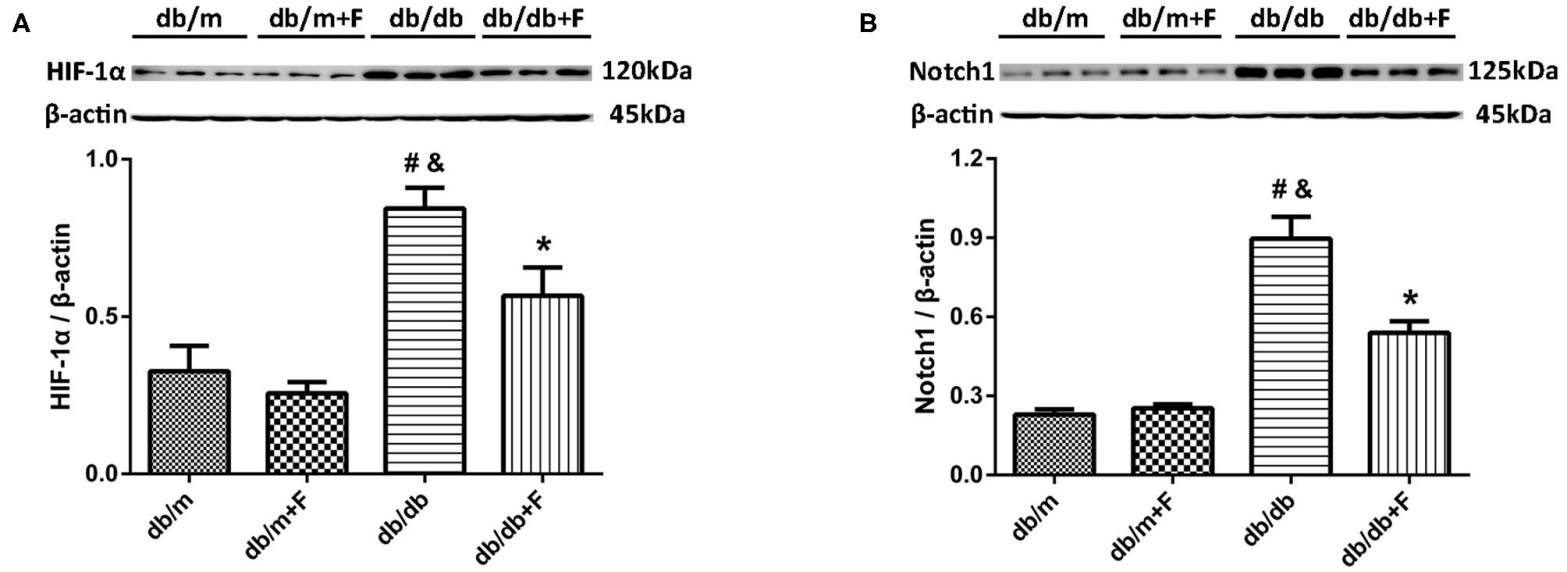
Hypoxia-inducible factor (HIF)-1α and Notch1 in mouse kidneys. **(A,B)** Representative photographs and quantification of HIF-1α **(A)** and Notch1 **(B)** in mouse kidneys measured by western blot. *n* = 6 mice/group. ^#^*P* < 0.05 vs. db/m group; ^&^*P* < 0.05 vs. db/m+F group; *P < 0.05 vs. db/db group. Db/m, db/m mice without fenofibrate treatment; db/m+F, db/m mice with fenofibrate treatment; db/db, db/db mice without fenofibrate treatment; db/db+F, db/db mice with fenofibrate treatment. Data are means ± S.E.M.

Endothelial dysfunction might cause tissue hypoxia and the upregulation of HIF-1α expression (9, 11). In our previous study, increased HIF-α in endothelial cells was documented to exacerbate renal fibrosis by upregulating Notch (10). In present research, HIF-1α and Notch1 were elevated in kidneys of db/db group compared with db/m and db/m+F groups, and were reduced in kidneys of db/db+F group compared with db/db group after fenofibrate treatment (Figures 6A,B).

### M1 Macrophage Phenotype

**Figure 7:**
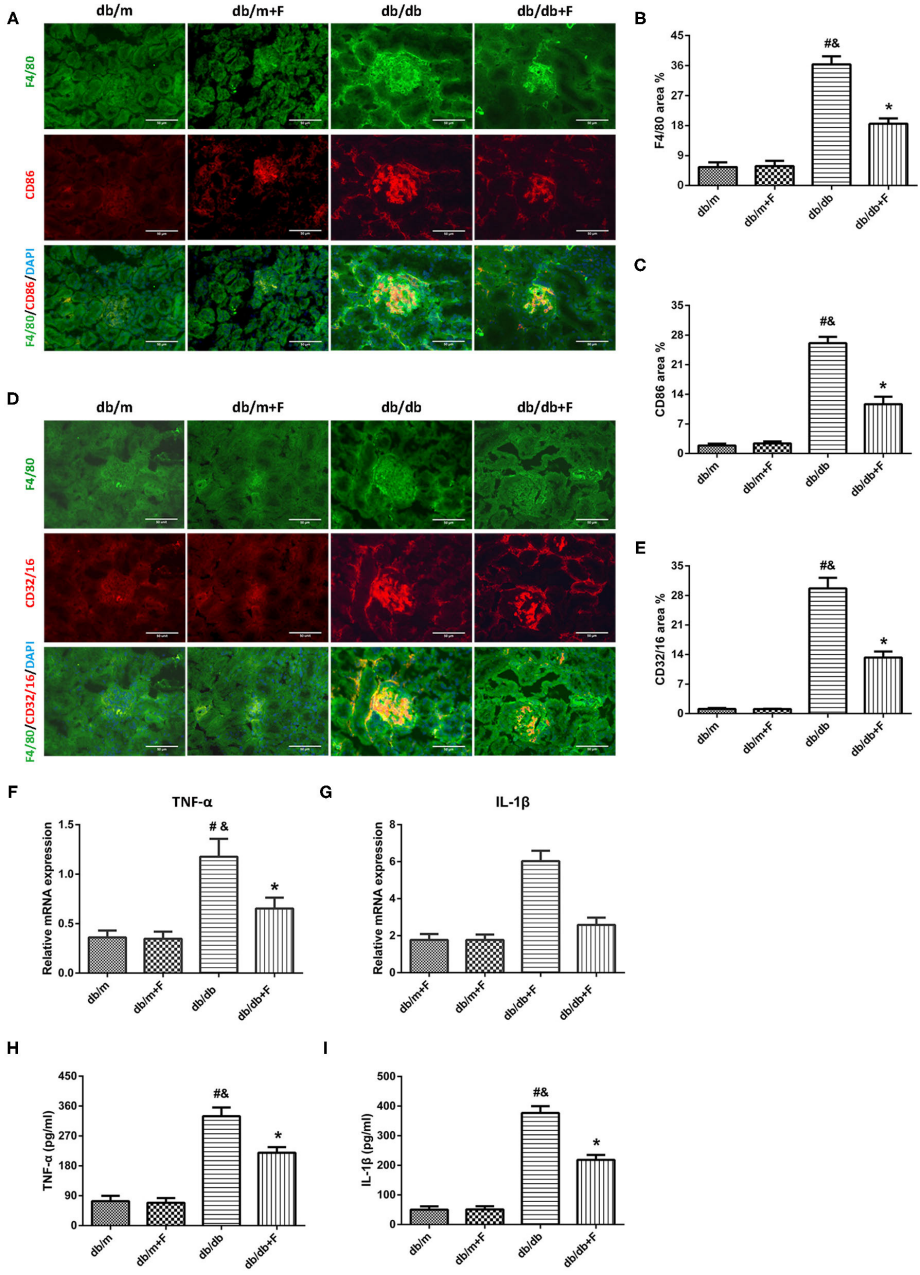
M1 macrophages in mouse kidneys and serum. **(A–C)** Representative photographs and quantification of co-staining of F4/80 and CD86 in mouse kidneys. **(D,E)** Representative photographs and quantification of co-staining of F4/80 and CD32/16 in mouse kidneys. **(F,G)** MRNA expression analyses of tumor necrosis factor (TNF)-α **(F)** and interleukin (IL)-1β **(G)** by quantitative reverse transcriptase PCR (RT-PCR). **(H,I)** Serum TNF-α **(H)** and interleukin (IL)-1β **(I)**. *n* = 6 mice/group. ^#^*P* < 0.05 vs. db/m group; ^&^*P* < 0.05 vs. db/m+F group; *P < 0.05 vs. db/db group. Db/m, db/m mice without fenofibrate treatment; db/m+F, db/m mice with fenofibrate treatment; db/db, db/db mice without fenofibrate treatment; db/db+F, db/db mice with fenofibrate treatment. Data are means ± S.E.M.

M1 macrophage polarization plays a critical role in fibrosis, which is regulated by endothelial cell function (18–20), associated with HIF-1α (22, 23) and controlled by Notch signal pathway (1, 15–17). Thus, we explored the M1 macrophage phenotype in mouse blood and kidneys. As shown in Figure 7, the immunostaining fraction of F4/80 was 5.55, 5.87, 36.38, and 18.35% in db/m group, db/m+F group, db/db group, and db/db+F group, respectively. The immunostaining fraction of CD86 was 1.88, 2.37, 26.09, and 11.63% in db/m group, db/m+F group, db/db group, and db/db+F group, respectively. Moreover, the immunostaining fraction of CD32/16 was 1.09, 1.05, 29.67, and 13.29% in db/m group, db/m+F group, db/db group, and db/db+F group, respectively. The co-staining of F4/80 and CD86 and the co-staining of F4/80 and CD32/16 showed that M1 macrophages were increased in kidneys of db/db group in comparison to db/m and db/m+F groups, whereas M1 macrophages was reduced in kidneys of db/db+F group compared with db/db group (Figures 7A–E). Similarly, mice in db/db group had higher cytokines associated with M1 macrophages, including TNF-α and IL-1β both in kidney measured by RT-PCR and in serum measured by ELISA, than mice in db/m and db/m+F groups, but these cytokines in kidney and serum were diminished in db/db+F group compared to db/db group (Figures 7F–I). These results indicated that M1 macrophage recruitment involved in the renal injury of type 2 diabetic mice, and fenofibrate suppressed M1 macrophage phenotype in type 2 diabetic mice.

## Discussion

In present study, we found that db/db mice presented higher UACR, more significant renal histological damage, and more significant podocyte injury than db/m mice, renal fibrosis and apoptosis were elevated in db/db mice compared with db/m mice, and the more significant endothelial dysfunction and the increased levels of HIF-1α and Notch1 in db/db mouse kidneys compared with db/m mouse kidneys. On the contrary, PPAR-α agonist fenofibrate decreased UACR, renal histological damage, and podocyte injury in db/db mice, and reduced fibrosis and apoptosis, ameliorated endothelial cell function, and depressed the expression of HIF-1α and Notch1 in db/db mouse kidneys. Importantly, we further demonstrated that type 2 diabetes led to promoted M1 macrophage recruitment in mouse kidneys, while fenofibrate treatment downregulated M1 macrophage recruitment in mouse kidneys of type 2 diabetes.

PPAR-α agonist fenofibrate is known as an important lipid-lowering drugs in clinical. Moreover, PPAR-α agonist fenofibrate has been proven to prevent DN and reduce urinary albumin in diabetic patients (26, 27) and in diabetic mice (29–32), which is independent of the effect on lipid-lowering. In current research, mice in different groups were similar in total cholesterol (TC). Body weight (BW), kidney weight (KW), food intake, blood glucose (GLU), insulin (INS), and triglycerides (TG) were increased in db/db and db/db+F groups compared with db/m and db/m+F groups. There were no differences in BW, KW, food intake, GLU, INS, and TG between db/m and db/m+F groups, and between db/db and db/db+F groups. Db/db group presented higher UACR than db/m and db/m+F groups, and db/db+F group had the significantly lower UACR level than db/db group. Diabetes causes glomerular mesangial expansion and glomerulosclerosis in kidneys, and leads to glomerular podocyte injury (41). In current research, these pathological changes were found in mouse kidneys of db/db group, while fenofibrate improved these injuries in mouse kidneys of db/db+F group. These results provided a strong evidence of PPAR-α agonist fenofibrate to prevent DN.

Fibrosis is an important pathological manifestation of DN (42), and apoptosis plays a critical role in the pathogenesis of DN (43). In current study, no significant difference of renal fibrosis area was observed between db/m and db/m+F groups. In contrast, there was a significant increase in the renal fibrosis area in kidneys of db/db group as compared to db/m and db/m+F groups. Consistent with the changes of the fibrosis fractional area, the expression of collagen I was significantly increased in kidneys of db/db group as compared to db/m and db/m+F groups. Moreover, apoptosis detected by TUNEL and apoptosis associated protein-cleaved caspase-3 in kidneys of db/db group were exacerbated compared with db/m and db/m+F groups. However, these renal fibrosis and apoptosis changes were ameliorated by fenofibrate treatment in db/db+F group compared to db/db group. These findings suggested that PPAR-α agonist fenofibrate prevented type 2 diabetes-induced renal fibrosis and apoptosis.

DN is one of the diabetic microvascular complications. Vascular endothelial dysfunction plays a crucial role in diabetic renal injury. Ang-2, an indicator of endothelial injury, can be induced by hyperglycemia in endothelial cells (44), and can regulate macrophage polarization (18–20). Our previous study has found that Ang-2 was increased in PHD2 ECKO mice, which was associated with the elevated HIF-1α and fibrosis in mouse kidneys (10). ENOS knockout mice with both type 1 and type 2 diabetes are sensible to DN in comparison to wild type mice with diabetes (6–8). ENOS provides the principal means by which NO is generated in the kidneys (3). Decrease in NO is the major cause of diabetic vascular complications, including DN (2). In our previous study, high glucose caused endothelial dysfunction with reduced NO generation and elevated ROS production in HUVECs (33); however, fenofibrate recoupled eNOS and promoted NO in HUVECs (34). In addition, our previous study found that fenofibrate improved arterial stiffness and CFVR in patients with hypertriglyceridemia (35). Our previous findings suggested that PPAR-α agonist fenofibrate improved endothelial function and vascular tone. The current study showed a significant enhancement of Ang-2, and a significant inhibition of p-eNOS/t-eNOS and NO in mouse kidneys of db/db group compared with db/m and db/m+F groups, but fenofibrate treatment decreased Ang-2, and promoted p-eNOS/t-eNOS and NO in mouse kidneys of db/db+F group. This was accompanied by a significant elevation of ROS formation in mouse kidneys of db/db group compared with db/m and db/m+F groups, which was suppressed by fenofibrate treatment in mouse kidneys of db/db+F group compred to db/db group.

Interestingly, recent evidence has indicated that HIF-1α is associated with endothelial function. However, the data have been inconsistent. There have been some studies suggesting that low HIF-1α expression might be correlated with endothelial dysfunction (45, 46). Controversially, most studies have indicated that the upregulated expression of HIF-1α was induced by tissue hypoxia due to endothelial dysfunction and deficient NO production (47), and the inhibition of eNOS by N-nitro-L-arginine methyl ester (L-NAME) promoted the expression of HIF-1α (9). Recent studies have proven that the inhibition of HIF-1 protected against diabetic renal injury (11). In our previous research, elevated HIF-α in endothelial cells enhanced renal fibrosis (10). Moreover, PPARα agonists reduced hypoxia-induced HIF-1α expression and activity in cancer cells (38), and improved ischemic retina diseases through decreasing HIF-1α in endothelial cells (36, 37). In present study, the level of HIF-1α was increased in kidneys of db/db group compared with db/m and db/m+F groups, and was decreased after fenofibrate treatment in mouse kidneys of db/db+F group compared with db/db group. It might be hypothesized that the elevated HIF-1α expression could reflect the hypoxia on db/db mouse kidneys, which might be caused by endothelial dysfunction, decreased NO production, and endothelial-dependent vasodilation dysfunction induced by hyperglycemia. PPAR-α agonist fenofibrate could improve endothelial function and increase the production of NO, and could consequently improve hypoxia and suppress the HIF-1α level in kidneys of type 2 diabetic mice.

Notch accelerates fibrosis and apoptosis in diabetic renal injury (14, 47). Recent studies have proven that Notch could be activated by HIF-1α (12, 13), and our previous study demonstrated that increased HIF-α in endothelial cells accelerated renal fibrosis through Notch activating (10). Additionally, upregulating PPAR-α could suppress Notch-1 signaling (39). In present research, Notch1 was enhanced in kidneys of db/db group compared with db/m and db/m+F groups, and fenofibrate decreased the expression of Notch1 in mouse kidneys of db/db+F group.

Macrophage polarization plays an important role in the development of renal fibrosis (1). The effects of macrophage polarization in the progression of DN have not been adequately defined. However, some studies have supported that macrophage polarization was strongly correlated with the pathological mechanism of DN (48, 49). Furthermore, there have been studies which demonstrated that the upregulation of M1 macrophage polarization accelerated renal fibrosis (1). It has been proven that endothelial function regulates macrophage polarization (18–20). Recent researches have documented that hypoxia exerted great effects on macrophage polarization, and there was a significant relationship between HIF-1α and M1 macrophage polarization (22, 23). Notch has also been proven to promote renal inflammation and regulate macrophage polarization (1, 15–17).

The co-staining of F4/80 and CD86 and the co-staining of F4/80 and CD32/16 showed increased M1 macrophage recruitment in kidneys of db/db group compared to db/m and db/m+F groups, and this recruitment was eliminated by fenofibrate in kidneys of db/db+F group compared with db/db group. Moreover, cytokines that were associated with M1 macrophages both in kidneys and in serum, such as TNF-α and IL-1β, were elevated in db/db group compared to db/m and db/m+F groups, and were repressed after fenofibrate treatment in db/db+F group compared with db/db group. These results indicated that M1 macrophage recruitment enhanced the development of DN in type 2 diabetic mice, while fenofibrate relieved M1 macrophage recruitment in type 2 diabetic mouse kidneys.

## Conclusions

In summary, M1 macrophage recruitment due to the upregulated HIF-1α/Notch1 pathway induced by endothelial cell dysfunction involves in type 2 diabetic mouse renal injury, and PPAR-α agonist fenofibrate prevents DN by reducing M1 macrophage recruitment through inhibiting HIF-1α/Notch1 pathway caused by the improved endothelial cell function in type 2 diabetic mouse kidneys.

## Data Availability Statement

The raw data supporting the conclusions of this article will be made available by the authors, without undue reservation.

## Ethics Statement

The animal study was reviewed and approved by Animal Ethics Committee of Beijing Chao-Yang Hospital, Capital Medical University.

## Author Contributions

XF and XG: design, experimentation, statistics, and article revision. SW: experimentation, statistics, and article revision. MH, ZS, HD, and HY: experimentation. GW: design, statistics, and article revision. All authors contributed to the article and approved the submitted version.

## Conflict of Interest

The authors declare that the research was conducted in the absence of any commercial or financial relationships that could be construed as a potential conflict of interest.
