# Supplementary material for: PPAR-α Agonist Fenofibrate Prevented Diabetic Nephropathy by Inhibiting M1 Macrophages via Improving Endothelial Cell Function in db/db Mice
Source: Front Med (Lausanne). 2021 Jun 29;8:652558. doi: 10.3389/fmed.2021.652558 (PMC8275839; doi:10.3389/fmed.2021.652558)

COLLAGEN I (130 kDa, rabbit)

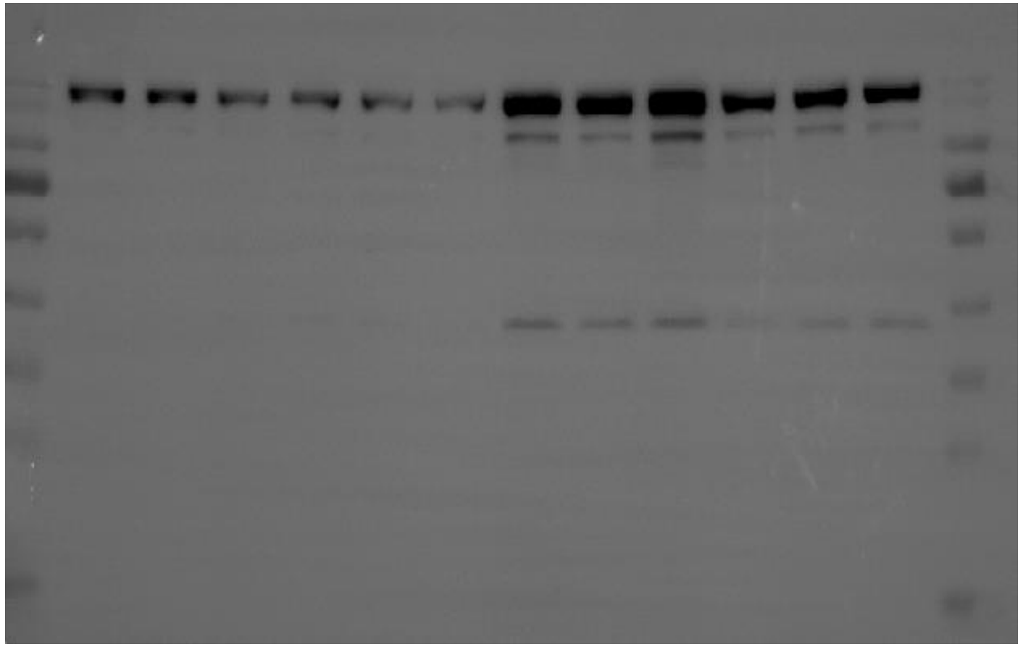

Podocin (34.4 kDa, rabbit)

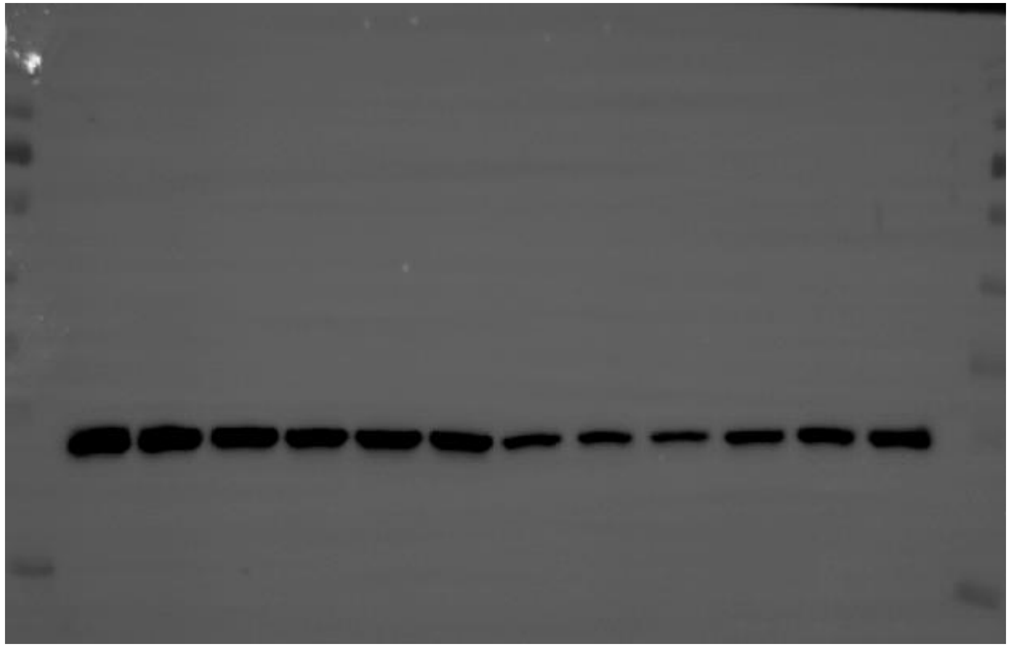

cleaved caspase-3 (31 kDa, mouse)

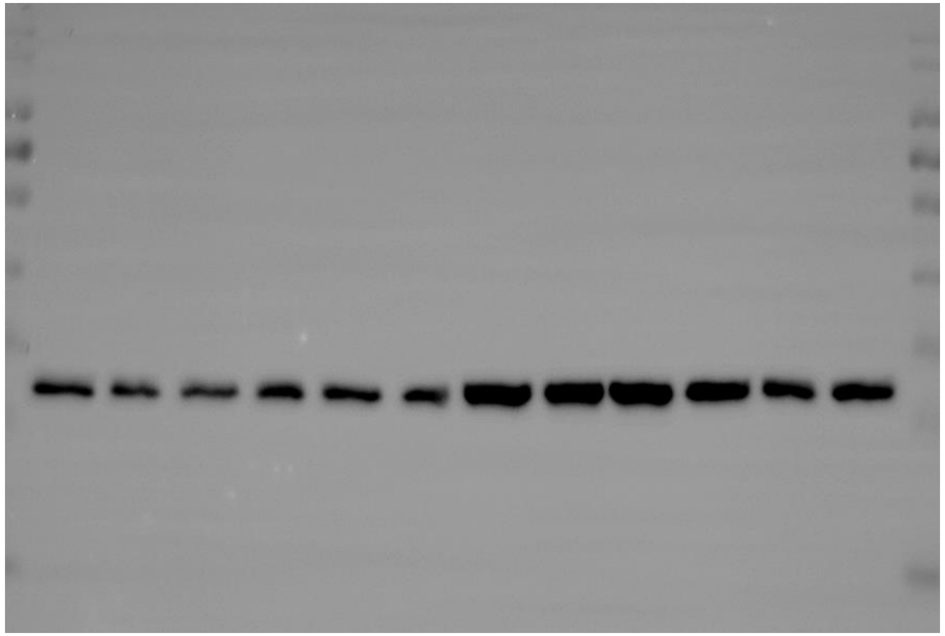

Ang-2 (62 kDa, mouse)

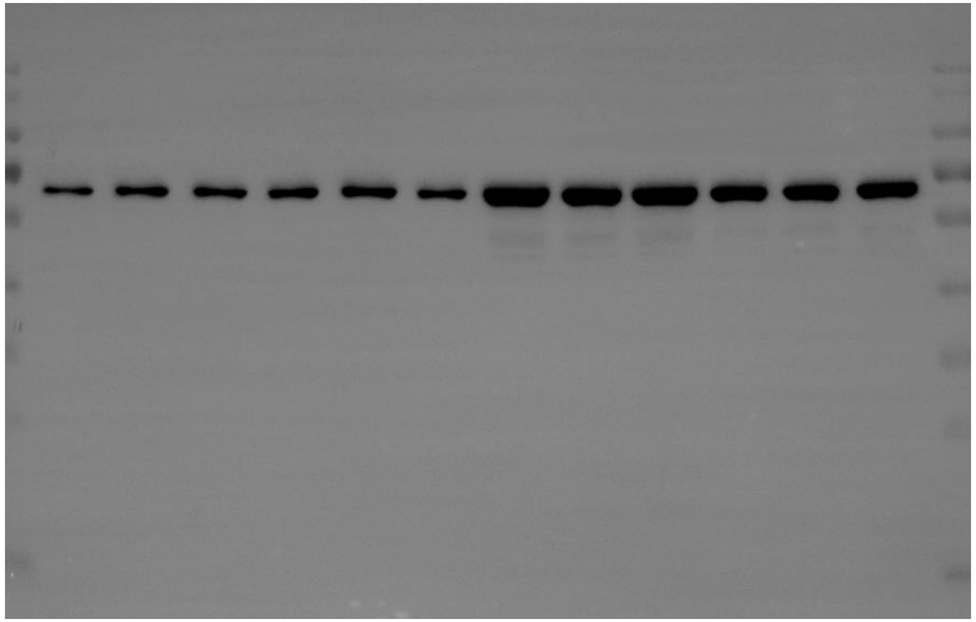

Notch1 (125 kDa, rabbit)

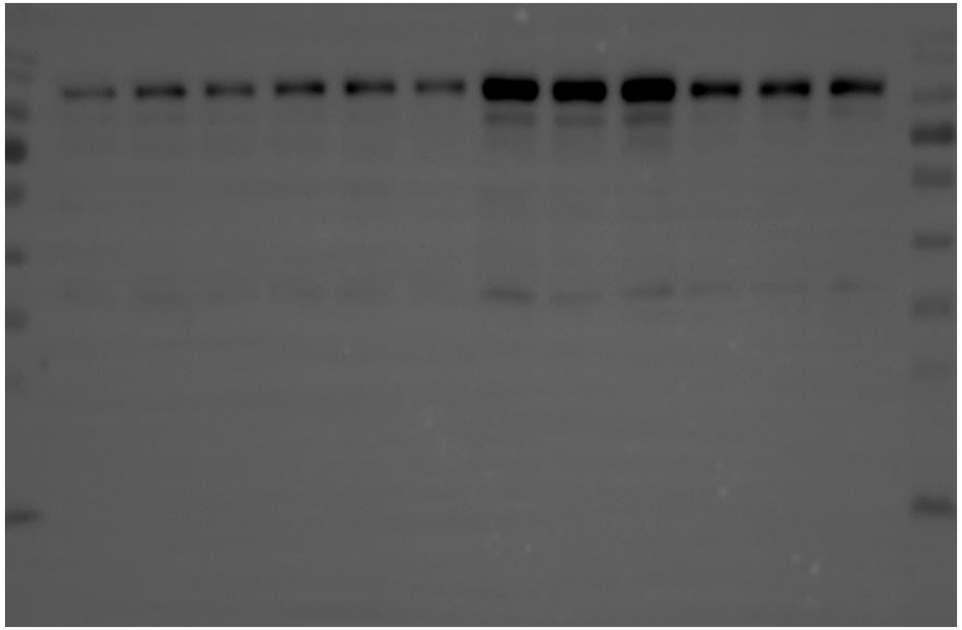

HIF1a (110 kDa, mouse)

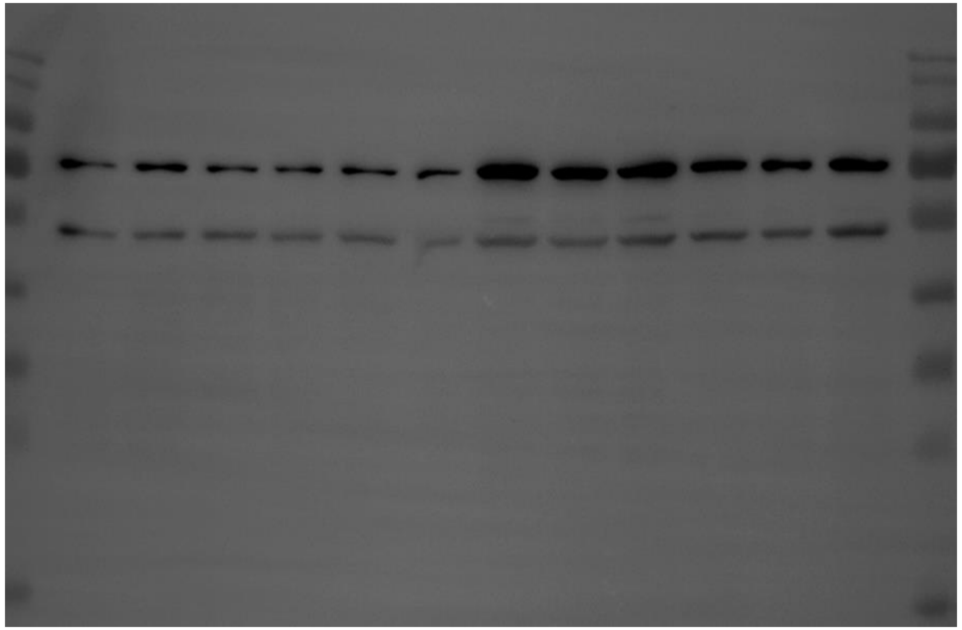

p-eNOS (140 kDa, mouse)

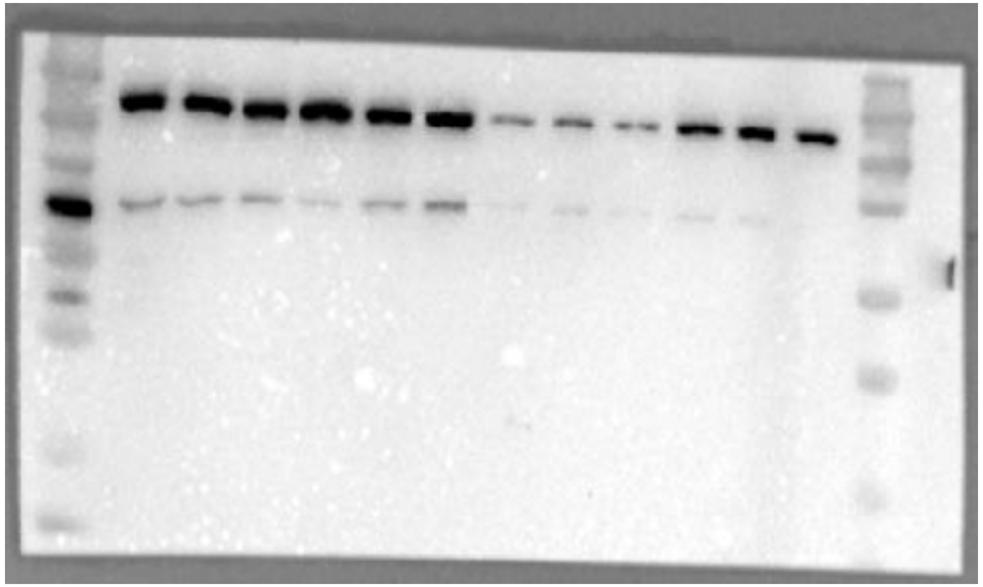

t-eNOS (140 kDa, mouse)

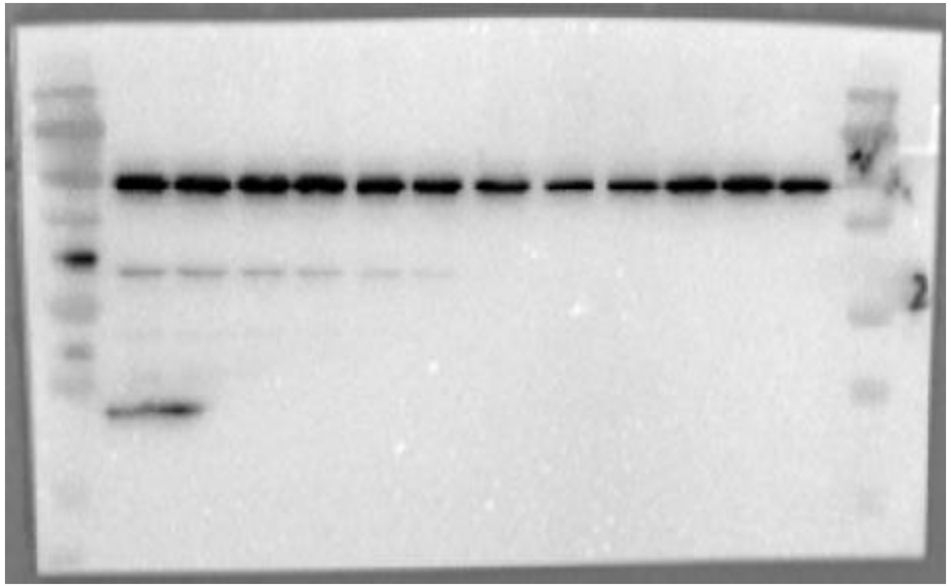

Supplement: Supplementary file 1 [file Image_1.PDF]
